# Tau’s Three-Repeat Domain and EFhd2 Co-incubation Leads to Increased Thioflavin Signal

**DOI:** 10.3389/fnins.2018.00879

**Published:** 2018-12-03

**Authors:** Irving E. Vega, Alexandra Sutter, Luke Parks, Andrew Umstead, Magdalena I. Ivanova

**Affiliations:** ^1^Department of Translational Science and Molecular Medicine, College of Human Medicine, Michigan State University, Grand Rapids, MI, United States; ^2^Michigan Alzheimer’s Disease Center, University of Michigan, Ann Arbor, MI, United States; ^3^Department of Neurology, University of Michigan, Ann Arbor, MI, United States; ^4^Biophysics Program, University of Michigan, Ann Arbor, MI, United States

**Keywords:** tau, EFhd2, amyloid, aggregation, Alzheimer’s disease, tauopathy

## Abstract

Aggregation of the protein tau is a pathological hallmark of Alzheimer’s disease (AD) and related disorders. However, the molecular mechanisms that lead to tau protein aggregation are still unclear. Previously, we showed that EFhd2 protein is associated with pathological aggregated forms of tau in AD brain. Further, immuno-gold analyses of purified tau aggregates showed that EFhd2 co-localized with filamentous tau structures. We demonstrated that EFhd2’s coiled-coil domain is required for its association with tau proteins. However, it is unknown the role that EFhd2 plays in tau aggregation. Here, we show that incubation of K19-tau with substoichiometric amount of EFhd2 promote the formation of amyloid structures *in vitro*. The result suggests that EFhd2 may play a role in the biogenesis of aggregated tau.

## Introduction

The accumulation of aggregated tau is a pathological hallmark of Alzheimer’s disease (AD) and related dementias (Chong et al., 2018). However, the molecular mechanisms that lead to tau aggregation are poorly understood. The accumulation of aberrant phosphorylated tau may promote the formation of specific conformations that render the protein prone to aggregation. Other posttranslational modifications, such as truncation, glycosylation, methylation, oxidation, have also been associated with the formation of tau aggregates (Mietelska-Porowska et al., 2014). Moreover, mutations on tau gene (MAPT) render tau proteins prone to aggregation (Bodea et al., 2016). Thus, molecular events that promote conformational changes may play a crucial role in the formation pathological tau species.

Tau’s microtubule binding domain, formed by three or four 18-amino-acid repeats, plays a crucial role in the formation of tau filaments. Specifically, *in vitro* studies reveal that three-repeat tau domain (K19) is sufficient for the formation of filamentous structures similar to paired helical filaments formed by full-length tau proteins (Friedhoff et al., 1998). Moreover, K19 tau can serve as a nucleation factor for the full-length tau and four-repeat tau segment (K18), but K18 cannot seed K19 tau (Friedhoff et al., 1998; von Bergen et al., 2005; Yu et al., 2012). However, most of these studies were conducted *in vitro* using excess of polyanions, such as heparin. Thus, the role of tau-associated proteins in the formation of tau aggregates and/or filaments is not well understood.

Previously, we identified the novel protein EFhd2 as a tau-associated protein in terminally ill JNPL3 mice, a tauopathy mouse model that expresses htau^P301L^ (Vega et al., 2008; Ferrer-Acosta et al., 2013). These results were subsequently validated in AD and FTDP17 postmortem brain. Further analyses, using immuno-gold electron microscopy and immunohistochemistry, demonstrated that EFhd2 was associated with filamentous tau and colocalized with aggregated tau in the somatodendric compartment in AD (Vega et al., 2008; Ferrer-Acosta et al., 2013). EFhd2 gene expresses an intrinsically disordered and highly conserved protein (from nematode to human) that is ubiquitous in neurons (Vega, 2016). In addition to the two EF-hand motifs required for its calcium binding activity, EFhd2 has other conserved structural features that may be relevant to its physiological and/or pathological roles. EFhd2 has an NT-terminal poly-alanine motif and C-terminal coiled-coil domain, both containing predominantly alpha-helical structures (Vega, 2016). We demonstrated that EFhd2’s coiled-coil domain is required for its association with tau proteins (Ferrer-Acosta et al., 2013). Taken together, EFhd2 may play a role in the biogenesis of pathological forms of tau proteins. Here, we show that incubation of K19 tau with substoichiometric concentrations of EFhd2 induces an increase in thioflavin-S (ThS) signal, suggesting the formation of amyloid structures. Additionally, we found that EFhd2’s coiled-coil domain is required for the increase in ThS signal. These results suggest that EFhd2 association with tau’s microtubule binding domain may promote the formation of amyloid structures.

## Materials and Methods

### EFHD2 Gene Cloning

Human EFHD2 wild type gene and C-terminal truncated mutant were produced by custom gene synthesis (Integrated DNA Technologies). The synthesized genes were subcloned into a bacterial expression vector pp-80L (5Prime Cat No. 2400850) in frame with an N-terminal 6x-Histidine tag, between BamHI (5′ site) and HindIII (3′ site) restriction sites. Plasmids were transformed into One Shot^TM^ BL21 (DE3) *Escherichia Coli* cells (Thermo Fisher Scientific, cat. # C600003). Plasmid DNA was extracted from colonies with positive expression of the expected protein molecular weight and subjected to DNA sequencing (GENEWIZ). Protein bands were also excised and subjected to trypsin in-gel digestion and tandem mass spectrometry analysis to confirm protein sequence.

### Recombinant Protein

A 50 mL LB/Ampicillin (50 μg/mL) pre-culture was incubated overnight at 37°C with constant shaking. Next day, 300 mL LB/Ampicillin was inoculated to 0.2 OD_600__nm_ using the saturated pre-culture and incubated at 37°C with constant shaking until the culture reached 0.5–0.7 OD_600__nm_. IPTG (0.5 mM) was added and the culture was incubated for 1 hr at 37°C. Cells were collected by centrifugation at 17,000 ×*g* for 10 min at 4°C and the pellet was resuspended in 10 mL of Lysate Buffer [1X PBS with 5 mM Imidazole (pH 8.0)]. After sonication on ice, the lysate was centrifuged at 14,000 rpm for 10 min at 4°C. Recombinant protein was purified using HIS-SELECT (Sigma, cat. # H0537-25 mL) beads (pre-calibrated in Lysate Buffer). The recombinant protein was eluted with 500 μL of 1xPBS containing 250 mM Imidazole (pH 8.0) and buffer exchanged by dialysis against 50 mM HEPES pH 7.0, 150 mM NaCl and 1 mM DTT. Concentrations were determined by BCA Protein Assay Kit (Pierce).

### Tau Purification

Tau construct K19, containing three repeats, R1, R3, and R4 was purified as described by Barghorn et al. (2005). In short, BL21 (Gold) were used as a host for expressing tau. Cells grown in 1 L LB broth were lysed in 10 ml of lyses buffer (50 mM Tris pH 7.4, 1 mM EDTA, 0.2 mM MgCl2, 5 mM DTT, 6 μl/ml saturated phenylmethylsulfonyl fluoride (PMSF), and 1 tablet Complete proteinase inhibitors (Roche) Cells were lysed in the high-pressure homogenizer (EmulsiFlex-B15, Avestin) and centrifuged at 30,000 ×*g* for 30 min. After adding NaCl to final concentration of 0.5 M, the supernatant was boiled for 20 min. Then the boiled sample was centrifuged at 20,000 ×*g* for 20 min. The supernatant was dialyzed against 10 mM Na phosphate pH 7.0 overnight. Next day the dialyzed protein was loaded on SP column (GE Healthcare), using 20 mM MES, pH 6.8, 50 mM NaCl, 1 mM EDTA, 1 mM MgCl2, 2 mM DTT, and 20 mM MES, pH 6.8, 1 M NaCl, 1 mM EDTA, 1 mM MgCl2, and 2 mM DTT, as elution buffer. After concentration, the protein was additionally purified on Superdex75 column equilibrated with 25 mM Na phosphate pH 7.4, 150 mM NaCl, and 1 mM DTT. Concentrations were determined by BCA Protein Assay Kit (Pierce).

### Thioflavin S (ThS) Binding Assay

Thioflavin S assays were carried out with 10 μM tau in reaction buffer containing 5 mM DTT, 150 mM NaCl, 50 mM HEPES pH 7.5, and 1 mM NaN_3_. Assays were performed in the presence of 5 mM EDTA and EGTA. As indicated, EFhd2 constructs (WT or ΔCT) were added to 0.2, 0.5, and 1 μM final concentrations. Prior to the assay proteins were filtered through a 0.22 μm filter. ThS was added to a final concentration of 20 μM. Teflon beads were added into each well of a Falcon 96-well plate (black/clear, flat bottom, Corning, 353219). Then 75 μl of sample were pipetted into each well. Plates were incubated at 37°C in a FLUOstar Omega (BMG Labtech Inc.) by shaking at 200 rpm using the ‘meander corner well shaking’ mode. Fluorescence was measured with gain set at 90%, an excitation wavelength of 440 nm and emission wavelength of 520 nm. Three technical replicates were measured per sample for a single ThS assay. Data shown are averaged over three independent experiments performed with different protein preparations.

## Results

**FIGURE 1:**
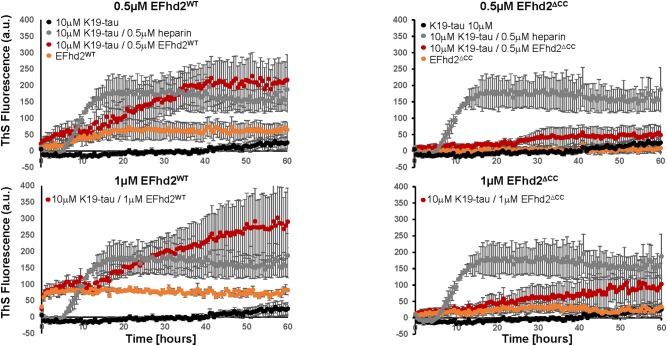
Co-incubation of tau and EFhd2 generates lead to increased Thioflavin S (ThS) signal. K19 tau segment was incubated with either wild type EFhd2 (EFhd2^WT^) or EFhd2 coiled-coil deletion mutant (EFhd2^ΔCC^). The graphs represent the data of 3 technical replicates per condition collected in three independent experiments. The standard error of the mean is illustrated after background subtraction using the fluorescence of the sample butter.

To determine if EFhd2 association with tau can promote the formation of amyloid structures, we incubated K19 tau with substoichiometric concentrations of recombinant EFhd2 protein. ThS fluorometry was used to assess the formation of amyloid structures. Full-length EFhd2 (EFhd2^WT^), at 0.2, 0.5, and 1.0 μM, was incubated with 10 μM K19-tau. As a positive control, we used K19-tau incubated with 2.5 and 1 μM heparin. K19-tau alone was used as a negative control. As expected, increase in ThS fluorescence was detected when K19-tau was incubated in presence of heparin, while tau alone did not show an increase in ThS emitted fluorescence (Figure 1). EFhd2^WT^ showed a higher background level of ThS signal that increased in a concentration dependent manner. Based on our previous studies, this result illustrates the capacity of EFhd2^WT^ for self-oligomerization. However, K19-tau incubated with substoichiometric concentrations (0.5 or 1 μM) of EFhd2^WT^ showed an increase in ThS fluorescence, but not 0.2 μM (Figure 1, data not shown). K19-tau incubated with heparin showed a rapid increase in ThS fluorescence that plateaued after 10 h. In contrast, K19-tau incubated with EFhd2^WT^ had a longer “lag-phase” where ThS fluorescence rapidly increased after 10 h of incubation, when both 0.5 and 1 μM EFhd2 were used (Figure 1). This result suggests that co-incubation of EFhd2^WT^ and K19-tau promote the formation of aggregates. This result also suggests that EFhd2^WT^ can associate with tau’s microtubule binding domain. To validate this observation, we incubated K19-tau with recombinant EFhd2 coiled-coil deletion mutant (EFhd2^ΔCC^) (Figure 1). The result shows a slight increase in ThS signal when EFhd2^ΔCC^ and K19-tau were co-incubated, in comparison to K19-tau co-incubated with EFhd2^WT^. Therefore, deletion of EFhd2’s coiled-coil domain reduces the effect observed when EFhd2^WT^ and K19-tau were co-incubated, suggesting that the association between EFhd2 and K19-tau is required to increase the formation of amyloid structures (Figure 1).

## Discussion

Here, we demonstrate, for the first time, that co-incubation of K19-tau with substoichiometric concentrations of EFhd2 proteins increases the formation of amyloid structures as detected by ThS fluorometry. The increase in ThS signal was detected when 0.5 and 1.0 μM were used and not with 0.2 μM of EFhd2^WT^, suggesting a concentration specific threshold to enhance the formation of amyloid structures (Figure 1, data not shown). Previously, we published that ThS binds EFhd2 *in vitro* in a concentration dependent manner (above 30 μM) (Ferrer-Acosta et al., 2013). Electron microscopy analysis of the samples demonstrated that EFhd2 proteins self-aggregate forming filamentous structures (Ferrer-Acosta et al., 2013). However, at base level, EFhd2^WT^ showed a higher ThS signal than EFhd2^ΔCC^, but both remained constant throughout the experiment. In contrast, K19-tau alone showed a slight increase in ThS signal after 40 h of incubation. These results indicate that co-incubation of EFhd2^WT^ with K19 tau increases ThS signal in EFhd2 concentration and coiled-coil dependent manner.

We previously showed that EFhd2 and tau co-localize at the somatodendric compartment and are found together in filamentous structures purified from AD brain (Ferrer-Acosta et al., 2013). Thus, it is plausible to hypothesize that EFhd2 may contribute to the formation of tau filamentous structures. The low concentration of EFhd2 proteins used in the experiments and the different phenomena observed when equal concentrations of EFhd2^WT^ and EFhd2^ΔCC^ were used makes molecular crowding an unlikely explanation for the increased of ThS fluorescence signal observed for samples of K19 tau co-incubated with EFhd2^WT^. Since ThS signal increased when both EFhd2 and K19 tau are co-incubated in comparison to both proteins incubated alone, we cannot discriminate between EFhd2 accelerating the formation of tau amyloid structures or that tau enhances EFhd2 amyloid formation. To distinguish between these two possibilities, more in-depth biophysical and structural analyses are needed. Nevertheless, here we show, for the first time, that EFhd2 and K19-tau co-incubation increases ThS signal. Further experiments are required to determine the pathological role of EFhd2 in tauopathies.

## Author Contributions

IV and MI conceptualized the experiments and wrote the manuscript. IV produced the recombinant proteins and analyzed the data. MI, AS, and LP purified recombinant proteins, conducted experiments, prepared figure, and analyzed data. IV and AU developed EFhd2 recombinant protein purification protocol and cloned EFHD2 human genes into bacterial expression vector.

## Conflict of Interest Statement

The authors declare that the research was conducted in the absence of any commercial or financial relationships that could be construed as a potential conflict of interest.
